# Supplementary material for: 3D bioprinting of an implantable xeno‐free vascularized human skin graft
Source: Bioeng Transl Med. 2022 Apr 21;8(1):e10324. doi: 10.1002/btm2.10324 (PMC9842062; doi:10.1002/btm2.10324)
Supplement: Supplementary file 1 — Appendix S1 Supporting Information [file BTM2-8-e10324-s005.docx]

**Supplementary information**

**Isolation of human keratinocytes and fibroblasts from foreskin tissue**

Normal human skin cells were obtained from donated foreskin samples. Foreskin samples were received in saline solution and were washed with phosphate buffered saline (PBS) containing antibiotics (Pen Strep - Gibco, Life Technologies). Samples were cleaned and fragmented prior to digestion with 4mg/mL dispase II (Roche, Life Sciences) overnight at 4ºC. The dermis and epidermis were mechanically separated from each other. The dermis was subsequently incubated with 1mg/mL Collagenase (Gibco Life Technologies) for 3 hours at 37ºC to induce detachment of the fibroblasts from the collagen. The epidermis was incubated with 0.05% trypsin for 15 minutes at 37ºC to promote cell dissociation. Fibroblasts obtained from dermis, and keratinocytes obtained from epidermis were centrifuged at 1500g for 3 minutes and plated with specific culture media.

**Isolation of human endothelial colony forming cells from cord blood**

EC were differentiated from EPC from umbilical cord blood. Collection of cord blood was performed immediately following elective cesarean section, and the blood was anticoagulated with heparin. Mononuclear cells were enriched by density centrifugation using Lymphocyte Separation Medium (MP Biomedicals) according to manufacturer's instructions. Total mononuclear cells from cord blood were then plated onto human plasma-fibronectin coated tissue culture plastic and were cultured in EC-Cult-XF medium supplemented with 2mg/ml heparin. Nonadherent cells were gently removed by washing after four days. Colonies of proliferating differentiated cells were typically noted on days 7–10.

**Isolation of human pericytes from placenta**

Human placentas were obtained after cesarean section of healthy full-term infants and stored at 4°C for up to 6 hours before cell isolation. An approximately 10cm×10cm×3cm central section of human placental tissue, near the insertion of the umbilical cord, was manually dissected and freed from the amniotic sac under aseptic conditions. The tissue was washed three times in PBS and visible thrombi removed by aspiration. Washed tissue was minced continuously for 8–10 minutes with surgical scissors, placed into a sterile flask with an equal volume of Hanks Balanced Salt Solution (Gibco) containing 3 mg/mL of collagenase type D (Sigma-Aldrich), and digested for two hours at 37°C on an orbital shaker at vigorous speed (180 rpm). The digested suspension was first passed through a 100-μm mesh filter (BD Falcon) to remove large vessel segments and fibrous tissue. The flow-through, containing microvessel fragments and single cells, was collected and passed a second time through a 40-μm mesh filter (BD Falcon). The 40-μm filter was then inverted over a 100-cm tissue culture plate and reverse-washed with EC-Cult-XF medium supplemented with 2mg/ml heparin to collect microvessel fragments. Plates containing microvessels were left undisturbed for seven days in a 37°C 95%O2/5%CO2 incubator in 8 mL of medium to allow microvessel attachment and PC outgrowth. On culture day 7, medium was carefully aspirated and replaced with 10 mL of fresh complete medium, without dislodging adherent microvessel fragments. By day 10, extensive microvessel outgrowth colonies could be visualized by light microscopy, at which point cultures were washed twice with PBS and given fresh complete medium. Medium was changed every 48 hours thereafter until cells reached confluence, usually between culture days 14 and 21. Primary outgrowth cells were collected by aspirating medium, washing twice with PBS, and treating with 0.1% trypsin (Invitrogen) until detachment. Following trypsinization, cells were collected, centrifuged at 200×g for 10 minutes, resuspended in complete medium. Cells were thereafter propagated by passing them 1:2 and used between passages 1 and 8.
