# Supplementary figures and images for: 3D bioprinting of an implantable xeno‐free vascularized human skin graft
Source: Bioeng Transl Med. 2022 Apr 21;8(1):e10324. doi: 10.1002/btm2.10324 (PMC9842062; doi:10.1002/btm2.10324)

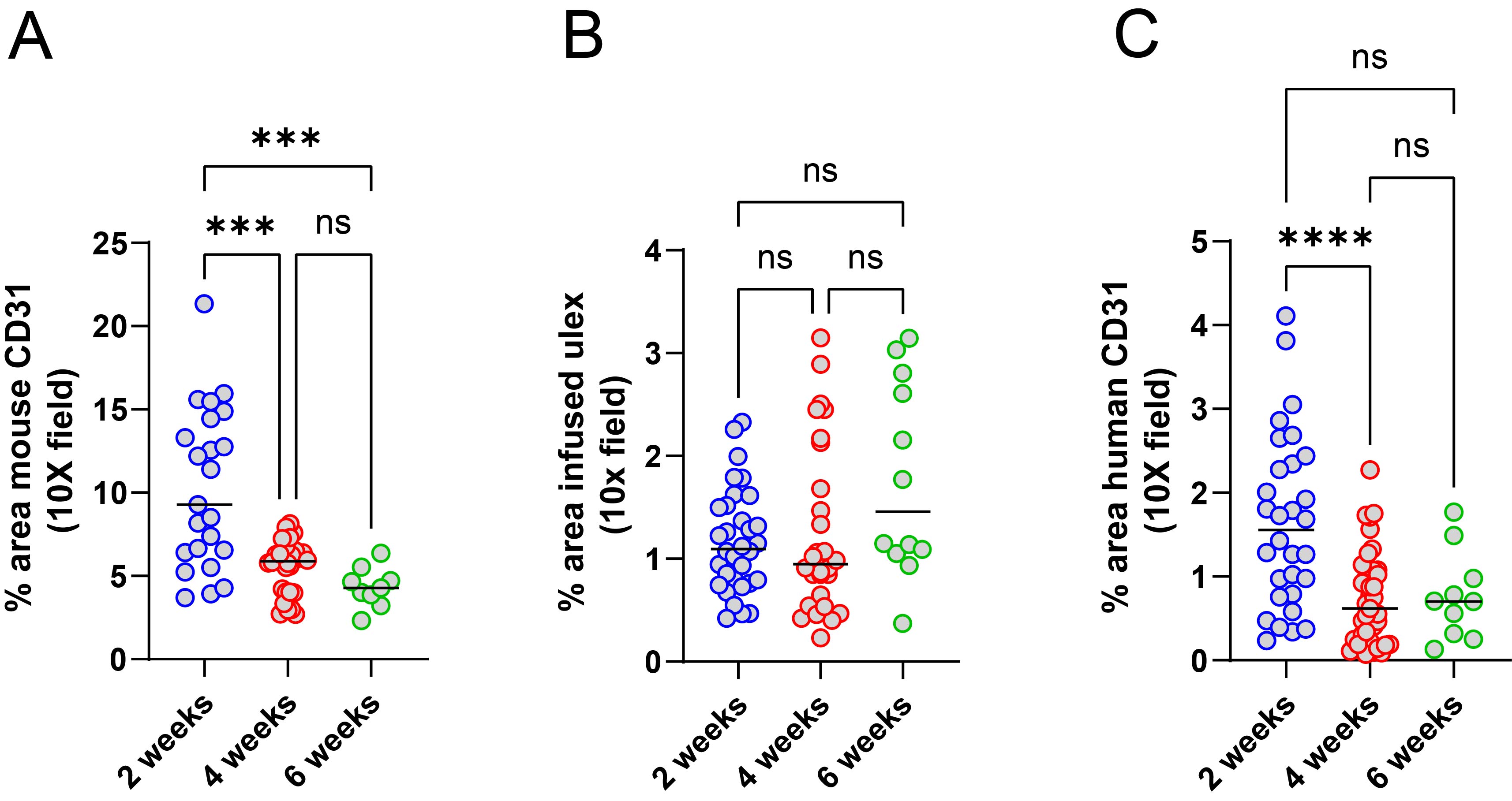

Supplement: Supplementary file 2 — Figure S1 Quantification of area of (A) mouse CD31, (B) infused ulex and (C) human CD31 at 2‐, 4‐, and 6‐weeks post‐implantation. (* indicates p < 0.05, ** indicates p < 0.01, *** indicates p < 0.001, **** indicates p < 0.0001, ns indicates p > 0.05; One‐way ANOVA method with Tukey post hoc comparisons). [file BTM2-8-e10324-s004.jpg]

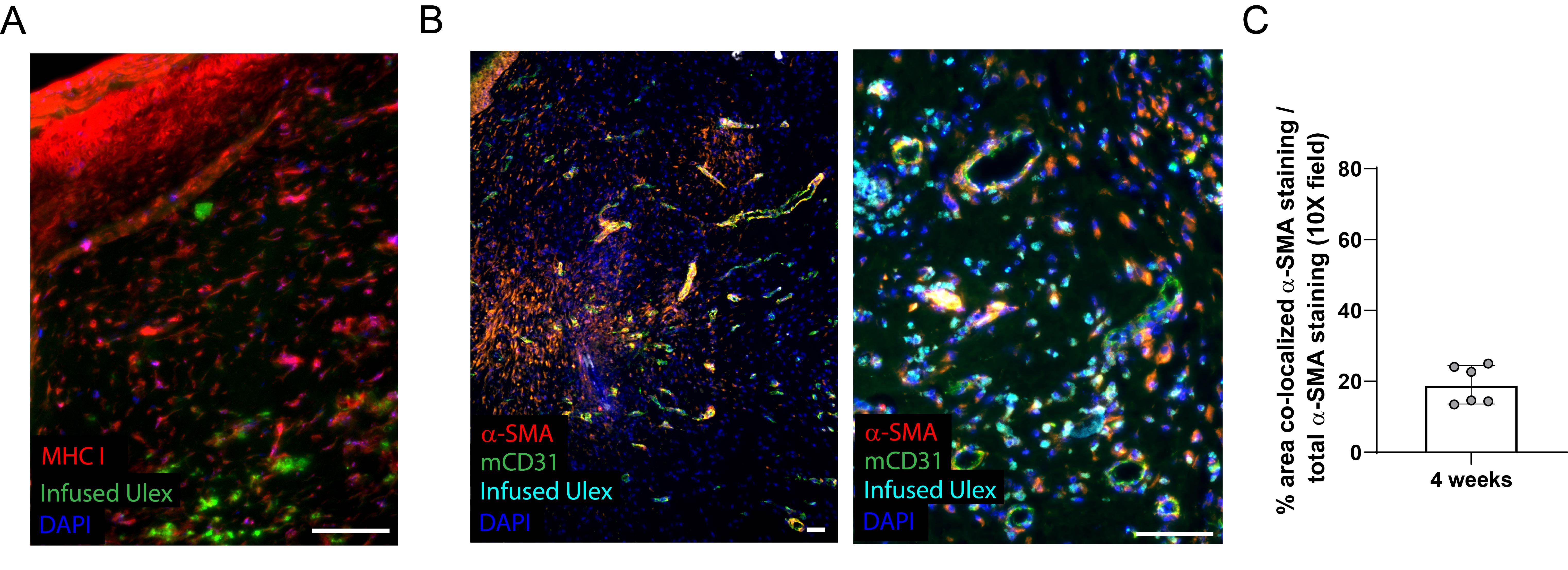

Supplement: Supplementary file 3 — Figure S2 (A) Human cells expressing MHC class I are present in the epidermis and dermis of xeno‐free grafts 4 weeks post‐engraftment. (B) Alpha‐SMA‐positive PCs associate with murine (green) and human ECs (cyan). (C) Quantification of PCs associated with vessels in xeno‐grafts 4 post‐implantation. Scale bars: 100 μm. [file BTM2-8-e10324-s003.jpg]

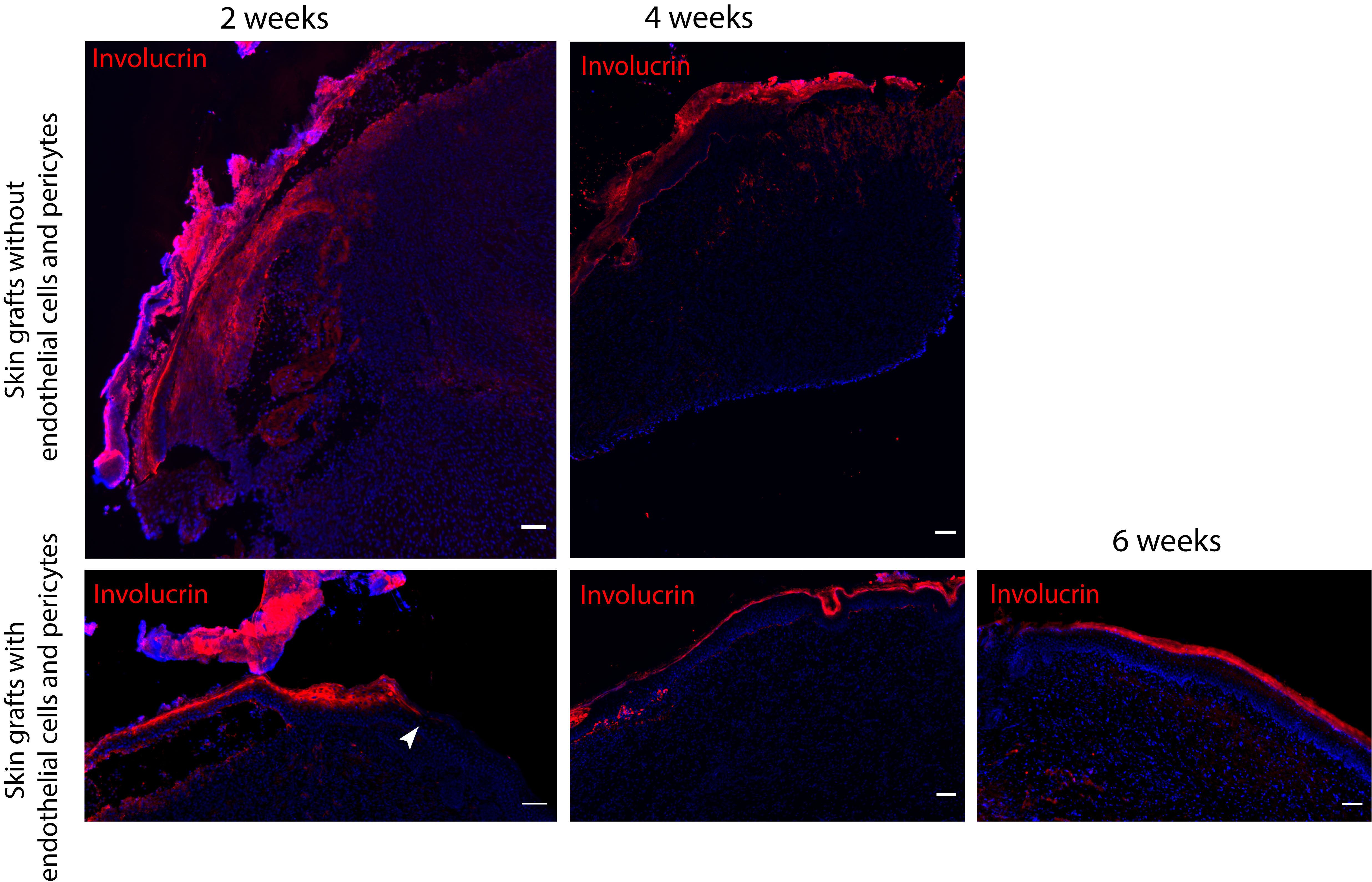

Supplement: Supplementary file 4 — Figure S3 Epidermis of bioprinted xeno‐free skin grafts with (lower row) and without ECs and PCs (upper row) is of human origin at 2‐, 4‐ and 6‐weeks post‐engraftment. Immunofluorescence staining of human involucrin showing that epidermis of 3D bioprinted skin is human. White arrowhead points to the edge of the wound. Scale bars: 100 um. [file BTM2-8-e10324-s002.jpg]

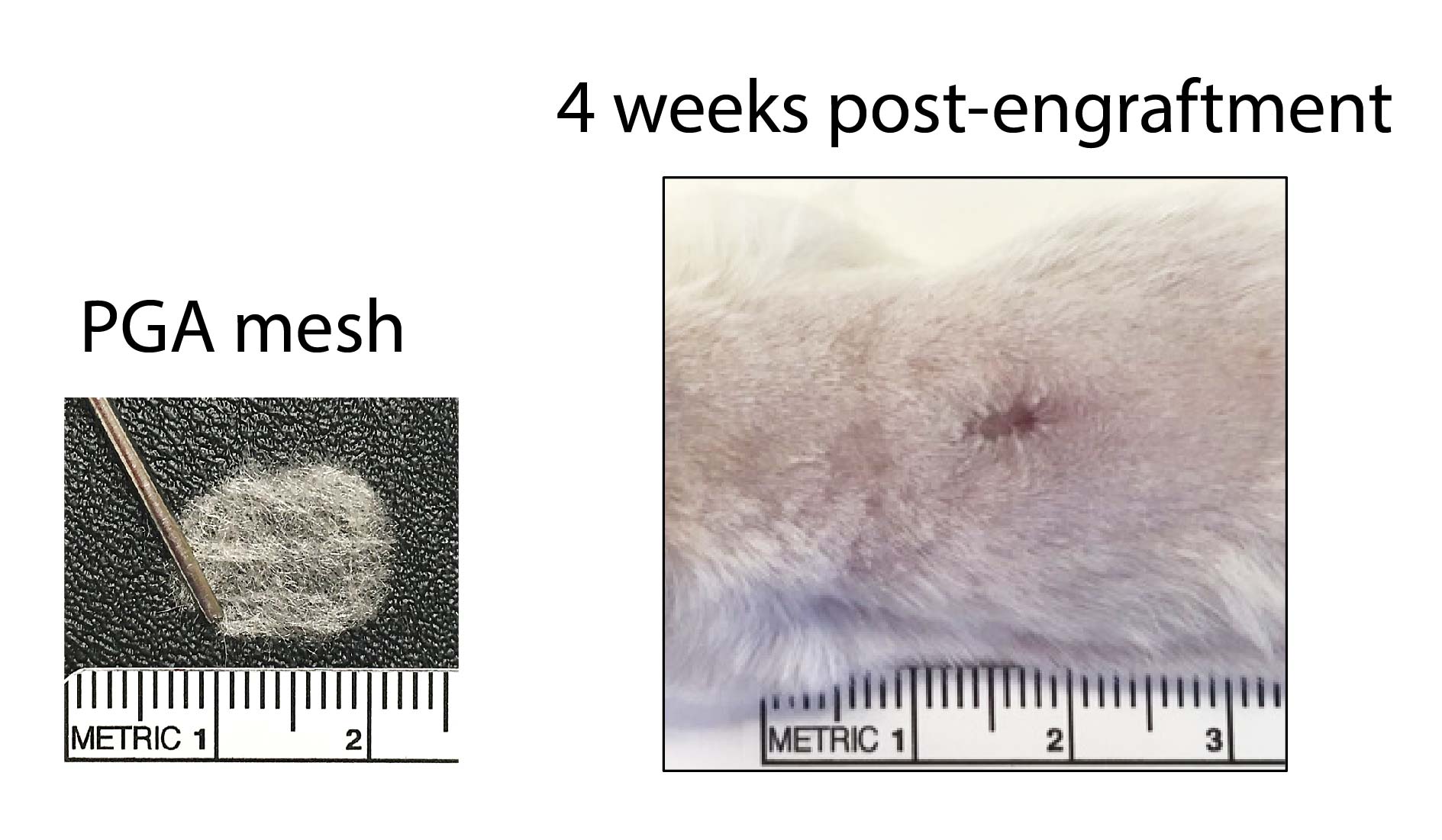

Supplement: Supplementary file 5 — Figure S4 Photographs of PGA mesh used in the fabrication of xeno‐free bioengineered skin grafts and graft at the time of harvest on the dorsum of SCID/bg mouse. [file BTM2-8-e10324-s001.jpg]
